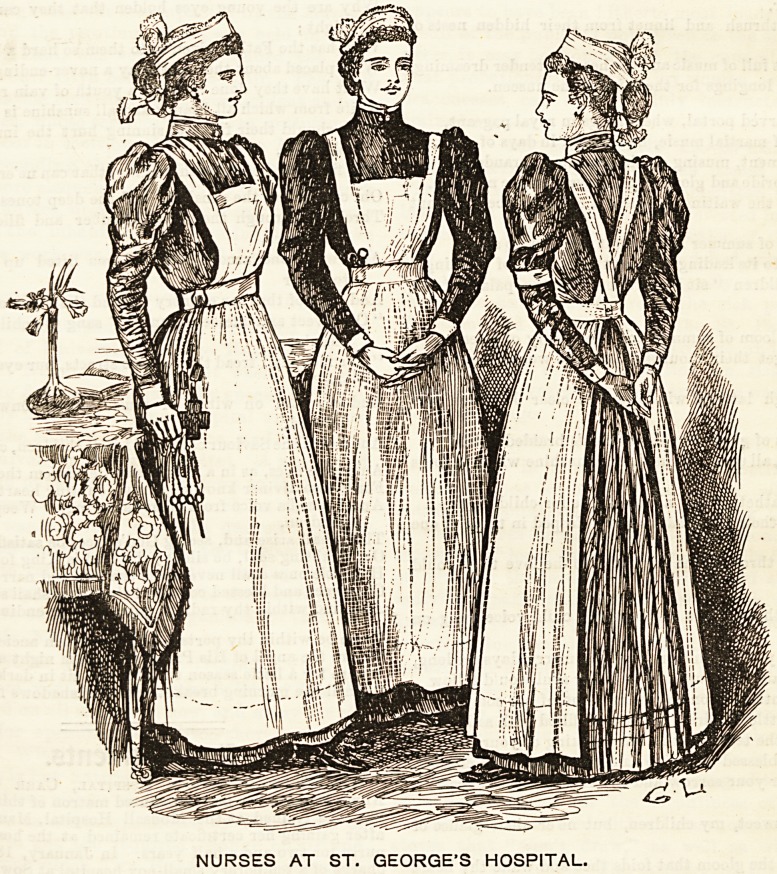# The Hospital Nursing Supplement

**Published:** 1895-06-15

**Authors:** 


### "The Hospital" Nursing Mirror.

Being the Extra Nursing Supplement of "The Hospital" Newspaper.

[Contributions for this Supplement should be addressed to the Editor, The Hospital, 428, Strand, London, W.C., and should have the word "Nursing" plainly written in left-hand top corner of the envelope.]

### News from the Nursing World.

### MEDALS FOR NURSES.

The English Government is still behindhand in its recognition of workers among the sick. This applies not only to physicians and surgeons, but to members of the nursing profession also. To many, a medal, recognising devoted services to the sick and others, would be a simple and sufficient proof of appreciation. In France the Government recognises this, and Madame Morel, who has given twenty-five years to the sick, having done valuable work during two small-pox epidemics, and Madame Dallery, who devoted herself to the sufferers from an epidemic of typhoid in Algiers, have both been awarded a silver medal by the French Government.

### NOT ALTOGETHER MONOTONOUS!

"I thought workhouse nursing was dull and monotonous, but your ward is quite lively this afternoon," commented a lady visitor. "Is there anything particular going on?" The charge nurse smiled as she glanced at the speaker. "Yes," she replied, "they have all had a shock, which has made quite a sensation." The visitor looked inquisitive, and nurse had to explain that the medical officer had admitted a patient, and directed that she must be washed between blankets. She was too ill to face the ordeal of the bath usually given downstairs by the wardswomen to new cases. The order was received without comment by the nurse, who had not been long installed, but she soon found herself beset with questions. "What *did* the doctor mean?" asked one. "Wash her between blankets, indeed!" The attendants (miscalled nurses) "had never heard of such a thing!" They wished to know where their duties would end if they had these things put upon them!" "In fact," said the modern charge nurse to the visitor, "if we had had two or three bad operation cases sent into the ward they would not have caused half the excitement of this one order, which, by the way, I had to carry out myself, as no one else would do so."

### INDULGENCE IN DRUGS.

Nurses ought to know better than other women the perils of morphia taking, but that such knowledge fails to make them shun danger is shown in the recent inquest on a nurse at the South-Western Hospital. She was found in an unconscious condition, and died in a few hours. It was suggested that she might have taken "an ordinary dose of morphia" to induce sleep, or that she might have had some given to her on a previous occasion for headache. The evidence pointed to her having taken a narcotic, but there was no probability that she contemplated suicide. Hence the deduction that she had taken morphia to induce sleep. For a nurse at any time to dose herself with morphia except under immediate medical advice is the height of folly, indicating also a condition of mind and body sufficiently serious. Already suffering from kidney disease, this poor nurse has paid with her life for her indiscriminate indulgence in drugs, and her sad fate adds a fresh warning to many previous ones given on this subject.

### ST. SAVIOUR'S HOSPITAL.

The concert recently given at Sir James Walker's under the patronage of H.R.H. the Duchess of Albany was well attended and successful. The artistes included Madame Alice Gomez, Miss Clare Putt, Mr. Leo Stern, Signor Simonette, and Mr. Brandon Thomas; the entertainment being in aid of St. Saviour's Hospital, Osnaburgh Street. This institution, attached to which is a beautiful chapel, now belongs to the Sisterhood of All Saints.

### NOVEL QUALIFICATIONS FOR NURSING.

Two candidates recently appeared before the Guardians at Stockport Union to apply for the post of nurse. One was a girl of eighteen and the other was twenty-one. The latter was asked by a member of the Board if she could sing, whilst another inquired whether she could teach Kindergarten? Both questions, it is reported in the local press, were greeted by laughter. Can it be possible the Guardians consider that they are likely to discover suitable nurses for sick inmates by such queries as these?

### NURSES' WORK, PAST AND PRESENT.

When educated women first took up hospital work the old untrained nurses were quite willing to own that "ladies were not above doing anything"; in fact, in their thirst to be useful the new comers were apt to let their zeal outrun their discretion. Unfortunately they were often unpractical and unhandy, therefore many details of the work were very badly performed, much time being wasted in learning how to put a quilt straight on a bed, and in deciding whether the outside as well as the inside must be included in washing basins. The woman of to-day, with a physique improved by outdoor exercise and healthy games, should accomplish with less fatigue to herself the mechanical duties of a nurse, and probably she does so, but she despises them. Theoretical instruction appeals to her vanity, and she thinks that a woman who masters even the most elementary anatomy wastes her talents in making the beds of helpless patients. Even the washing of dressing mackintoshes is sometimes thought beneath the dignity of the girl who talks glibly of germs whilst failing to realise how much she can do to destroy them by faithfully carrying out the details she despises as trivial.

### NURSING IN HOLLAND.

A welcome visit was paid in the spring by Her Majesty the Queen Regent of the Netherlands to the Wilhelmina Hospital of Amsterdam. The hospital put on its gayest appearance on the occasion, and Her Majesty was received by Dr. P. Place, the president of the committee, who in well-chosen words assured the Royal visitor that her wishes for its usefulness and prosperity, expressed when she laid the foundation-stone, had been well fulfilled. The Queen was then conducted over the hospital, visiting first the different wards, for whose inmates she had thoughtfully brought a quantity of flowers, an attention gratefully appreciated by the patients. Afterwards the kitchens and washhouses were inspected, the Royal visitor showing great interest in all the domestic arrangements. Her Majesty declared herself exceedingly pleased and impressed by the condition of the hospital. The new Protestant Hospital at Nijmegen, opened last May, has been planned with due regard to modern requirements; it is entirely dependent on voluntary contributions. The directors of the Sophia Sea-bathing Institute for Children at Scheveningen have acquired an extensive piece of ground as an addition to their present premises to be used as a playground for the children.

### TRAINING IN FINLAND.

The nursing staff at the Surgical Hospital at Helsingfors is composed of a matron and twelve staff nurses. The number of probationers under each nurse varies according to the number who happen to be undergoing training, but there are generally from twenty-eight to thirty at the hospital. They live in their own homes, coming to the hospital in the morning, returning home for dinner, and attending again at the hospital until the evening. A certain number of the more advanced are told off for night duty, which is undertaken exclusively by probationers, over whom the present system admits of no control as to hours of rest during the day. There is also the risk of their importing infectious diseases into the hospital. As there is no night superintendent the care of the patients at night must often be very insufficiently carried out. This is the only hospital in Finland where a regular course of training has been inaugurated and certificates given. The course lasts for a year. No fees are paid, but the services of the probationers belong to the hospital for that period. Lectures are given by the resident assistant surgeon and matron. After instruction for five months in the wards probationers have to attend operations for one month, then out-patients one month, and to go to a medical hospital for three months, an ophthalmic hospital one month, and a lying-in hospital one month. Those who pass their examinations best are generally chosen for a month's extra service in the wards, taking the place of the staff nurse whilst the latter is on her holiday. For this work no payment is made, the practice being considered advantageous to them, and receiving due mention in the certificate. The nurses as yet wear no regular uniform; cotton dresses and large aprons are, however, the rule. Caps, not being compulsory, are worn by only a few. The matron received her principal training in Berlin and Vienna. She takes the keenest interest and pride in the hospital, and by her kindness and courtesy advances its interest in every way.

### NURSES AT SAN FRANCISCO.

The Board of Lady Managers continue indefatigable in forwarding the good work of the Children's Hospital at San Francisco, and they are now contemplating the possibilities of adding to it a new wing. This will provide accommodation for incurable children, and will thus relieve pressure on the beds already in existence. A training school for nurses is attached to the hospital, and the establishment of a properly equipped nurses' home is urgently demanded. Physicians and lady managers are agreed on the need for proper recreation rooms, dormitories, &c., being provided without delay in a separate building. It seems probable that the home will be seriously discussed at an early date now that public attention has been drawn to the necessity for the proper housing of the nursing staff. Miss Elsie Wallace is the superintendent of nurses, and the badge chosen by the school consists of a gold pin with a diagonal band of white enamel bearing a red enamel cross.

### THE TRAINING OF DEACONESSES IN U.S.A.

A record of the subjects which are studied during the two years' preliminary course of training undergone by the episcopal deaconesses in New York gives three months' practical work in St. Luke's Hospital as the completion of the prescribed preparation. The foregoing instruction includes Old Testament history and literature; New Testament with theology and Church history, with a view to a thorough knowledge of the early Church, and especially its history in America, the liturgies of the Prayer Book, the Greek Testament, and the art of teaching; hygiene, physiology, and the care of children!

### NEW WORK FOR WOMEN.

America, which has acted as the pioneer for most new movements for the employment of women, has now opened the field of architecture to them. Fifteen tenement houses in New York are to be built after the plans of two women architects, who have devoted special attention to solving the problems involved in the erection of tenement house buildings. Women have shown capacities as sanitary inspectors, and in all the smaller details where health is concerned are especially fitted to take an active part, therefore there is no reason why they should not make very successful architects of hospitals and other buildings requiring special attention from a sanitary point of view.

### SHORT ITEMS.

The sick and injured soldiers from the Chitral Relief Force at the Base Hospital, Peshawur, have been tended by seven of the Indian Army Nursing Sisters.—It has been decided to secure the services of a Queen's nurse for Innerleithen.—The Camborne Nurse Fund is not prospering financially. According to the local press, there is a debt of nearly £100, a special collection made by ladies to meet this deficit resulting only in £21.—The Chester Guardians intend to ask the sanction of the Local Government Board to the appointment of two untrained women as nurses.—At Cadoxton Barry athletic sports were recently held and largely attended. The profits of the fête will substantially benefit the Cottage Hospital and Nursing Association.—A bazaar in aid of the local Nursing Institute was opened last week by the Duke of Rutland in the grounds of Belvoir Castle.—The Guardians have decided to appoint a trained nurse at the workhouse at Conway.—It transpired at a recent Board meeting at Lewes that the portress is expected to add night nursing to her other duties, and also (according to the local press report) the bathing of the female tramps is considered to be the cook's work!

### Elementary Anatomy and Surgery for Nurses.

By W. McADAM ECCLES, M.B., M.S., F.R.C.S., Lecturer to Nurses, West London Hospital, &c.

### XXI.—THE RESPIRATORY SYSTEM.

ALL human beings in common with the higher animals require to have their blood aërated in order that oxygen may be conveyed to the tissues. Therefore a special apparatus is found in the body whereby the air may be brought into near proximity with the blood. This is termed the respiratory system. It consists essentially of two hollow vesicular organs termed the lungs, and certain tubes which convey the external air into these. We can breathe in the atmosphere either through the nose or through the mouth, the former being the proper passage. The nose, therefore, besides being the seat of the organ of smell, is also a part of the respiratory system. The air entering the nostrils by the anterior nares traverses their cavities, which are separated from one another by the septum of the nose, and passes into the naso-pharynx or upper part of the pharynx through the posterior openings of the nose, the posterior nares. It then reaches the upper opening of the larynx or the organ wherein the voice is produced.

FIG. 30.—FRONT VIEW OF LARYNX AND TRACHEA.

a, Hyoid bone; b, Thyroid cartilage; c, Cricoid cartilage; d, Trachea; e, Bronchi.

The larynx consists of three single fairly large cartilages, and three pairs of much smaller ones forming together a framework, the parts of which are movable one on another. (See fig. 30.) The largest of these cartilages is called the thyroid cartilage, and can be felt in the middle line of the neck, just below the hyoid bone. In males it forms a very distinct prominence, often called Adam's apple. The two lateral halves of this cartilage unite in front, but are widely separated behind. The cricoid cartilage is situated just below the thyroid, and in shape somewhat resembles a signet ring, being the only complete ring of cartilage found in connection with the respiratory passage. It is narrow in front and broad behind, being surmounted posteriorly by a pair of very important but small pyramidal cartilages named the arytenoid. To the anterior and inferior angle of each of these is attached a fibrous band known as a vocal cord, which is fixed by its other end in front to the posterior surface of the thyroid cartilage. The opening between these two bands is called the rima glottidis, or chink of the glottis. Above the anterior attachments of the vocal cords will be seen a leaf-shaped cartilage which in swallowing is said to close the upper opening of the larynx, and so prevent food from entering it. This is spoken of as the epiglottis.

The larynx is followed by the trachea or windpipe, which consists of a series of cartilaginous rings incomplete behind, but united together by fibrous tissue which also fills in the open space posteriorly. Behind the trachea lies the œsophagus. On either side of the lower part of the larynx and the upper part of the trachea lies a solid gland-like organ, but without any duct, the two halves of which are united across the middle line over the third and fourth rings of the trachea by an intervening portion called the isthmus. The whole organ is called the thyroid body or gland. In quite young children another similar body, the thymus, lies in front of the lower part of the trachea. Neither of these organs has any immediate association apart from approximation with the respiratory system, but are merely mentioned here for convenience.

After a length of about four and a half inches the trachea bifurcates into two short tubes, the bronchi, which resemble the trachea in their general structure. The right bronchus is rather larger and more horizontal than the left. (See fig. 31.) Each of these tubes divide and sub-divide into smaller and still smaller vessels, which penetrate into all parts of the lungs, and ultimately at their terminations form groups of air cells surrounding the walls of which are very numerous capillaries of the pulmonary arteries, thereby the blood is brought into close relationship with the air in the vesicles.

The lungs will thus be seen to be large spongy elastic bags, which fill the thoracic cavity accurately one on either side, with the exception of the space occupied by the heart, its great vessels, and the œsophagus. The lungs are covered with a smooth, shining membrane—the pleura—which also lines the inner surface of the chest wall as well as the upper surface of the diaphragm; the portion covering the lungs is called the visceral pleura, that lining the chest walls is called the parietal pleura. The space between these two portions is the pleural cavity, which is lubricated with a little serous fluid, so in the movements of the chest in respiration the lungs glide on the thoracic walls without friction. The right lung, the larger of the two, has three lobes, whereas the left is the smaller, owing to the space occupied by the heart, and has only two lobes. The upper conical part of each lung is called the apex, while the lower broader part resting on the superior surface of the diaphragm is termed the base. Both lungs are convex externally, but concave internally, on which side about half way between the apex and the base will be found the root of the lung as it is named, which is composed of the pulmonary artery, the pulmonary veins, a bronchus with its blood-vessels, as well as lymphatics and nerves.

FIG. 31.—BACK VIEW OF LARYNX AND TRACHEA.

a, Hyoid bone; b, Epiglottis; c, Thyroid cartilage; d, Arytenoid cartilage; e, Cricoid cartilage; f, Trachea; g, Bronchi.

### Macclesfield Infirmary.

In the recent reports of the committee meetings at Macclesfield Infirmary very little mention has been made of Miss Wingfield, the matron's, personal endeavours at reforming the nursing department there. By her influence, we are told, an unused block was converted into convenient nurses' quarters in place of the noisy rooms off the corridors in which they were formerly accommodated. Miss Wingfield also abolished the custom of night and day nurses sharing the same apartments. As probationer, nurse, and matron, Miss Wingfield's own record appears to have been a good one; and at Ayr, where she held the position of matron in the County Hospital, she was highly appreciated, and she had much local encouragement in organizing a model nursing staff.

On several recent occasions it has been asserted that Miss Wingfield took a holiday from March till June last year when she entered on her duties at Macclesfield. So far from this being the case we learn that she spent that period in hard work at Ayr. When she found it necessary to delay her departure thence, in consequence of an accident which invalided another official, we believe she offered to relinquish her claim to the Macclesfield post. Her testimonials were, however, so exceptionally good that the committee expressed themselves willing to wait until she was free. The loyalty of her probationers and the affection and gratitude of the sick poor for whom so much has lately been done, show that up to the time of her illness, Miss Wingfield spared neither time nor strength in their service. It will be indeed disastrous if the committee allow things to lapse into the old ways, and if the period of training should be reduced. Some of Miss Wingfield's letters may rightly be accounted injudicious ones; but they were apparently written under provocation, and the good work already accomplished by her, as well as her ill-health at the time of writing, should secure more courteous treatment. The sympathy expressed by all classes at the severance of her connexion with the infirmary at Macclesfield cannot fail to be gratifying. "God bless you, you saved my child's life by what you did for him," exclaimed a tearful mother one day in the street as she seized hold of the matron's hand. Other incidents all tend to show that Miss Wingfield had endeared herself to many. It is a matter for regret that work so well begun should be thus interrupted.

### Our American Letter.

From Our Own Correspondent.

The school connected with the John Sealy Hospital, Texas, is maintained by the ladies of Galveston, the city in which the school stands. Twelve nurses are trained there, and more would be taken if less difficulty existed in the district in getting suitable women to undergo the course of instruction. Four classes of graduates have successively earned their diplomas at this school.

The nurses' club associated with the St. Peter State Hospital, Minn., held its anniversary meeting the other day, and a very pleasant evening was enjoyed. The guests inspected the library and reading room, which have been recently furnished and formally opened. The society, which has been established about a year, aims at stimulating professional feeling, promoting general culture, establishing a nurses' library, and furnishing the members with social entertainments. The library has been furnished by the members, and over a hundred volumes have already been collected, fifty nurses having joined the club since its opening.

Great excitement pervades the American nursing world with regard to the great question of a national pension fund. Steps have been taken in Philadelphia to formally promote some scheme, but the heads of the training schools in other cities remain strongly averse to what they consider an attempt to pauperise members of their profession. They maintain that there are plenty of existent investments where nurses can deposit their savings, and that this suffices. We are not ourselves aware that there is much proof that these deposits are made or that nurses have made provision for their future in America.

In the June number of *The Trained Nurse* it is announced that Miss Sophia F. Palmer will take future charge of the department. "Editorially Speaking," she is a trained nurse and a member of the Superintendents' Association. The same number of our American Nursing Journal contains an account of a nurses' home which has proved an undeniable success. Planned a year ago by the Nurses' Association at Baltimore, it is now working on most practical lines. Nurses furnish their own rooms, and take care of them, the bed and bedding alone being provided. Fifteen nurses entered in September, paying five dollars each per month in advance. At present they do their own cooking when at the home between cases, but as the scheme expands it is designed to have a restaurant and other conveniences.

Private nurses in England would, we imagine, hardly countenance these primitive methods, but their sisters in America have suffered so much inconvenience from the absence of permanent headquarters that they are exceedingly pleased with the outlook of the establishment which they have formed and worked for some months with unqualified success.

### Notes and Queries.

The contents of the Editor's Letter-box have now reached such unwieldy proportions that it has become necessary to establish a hard and fast rule regarding Answers to Correspondents. In future, all questions requiring replies will continue to be answered in this column without any fee. If an answer is required by letter, a fee of half-a-crown must be enclosed with the note containing the enquiry. We are always pleased to help our numerous correspondents to the fullest extent, and we can trust them to sympathise in the overwhelming amount of writing which makes the new rules a necessity. Every communication must be accompanied by the writer's name and address, otherwise it will receive no attention.

### Queries.

(158) *Foot Reform.*—Can you give me the name of the foot reform company?—*Nurse.*

(159) *Probationer.*—Is nineteen a good age to enter a hospital for training?—*Juvenile.*

(160) *Registration.*—Can a nurse be registered if she has had her training in more than one hospital, and would three months' training at Queen Charlotte's Hospital be counted in the three years to become registered?—*Nurse D. F. D.*

(161) *Vaccination.*—My child is two months old, I do not wish to have him vaccinated. Is there any society in London which would help me in this matter, and what will it cost?—*Anxious Father.*

(162) *Probationer.*—Could you please let me know who I ought to apply to for particulars as to entering a hospital as a probationer; where probationers are paid preferable.—*Enquirer.*

(163) *Probationer.*—Could you inform me how I could obtain a post as probationer most quickly in a general hospital for not less than two years' training? I would give three months' time.—*E. E. P.*

(164) *Male Nurse.*—Will you kindly tell me if there is a training school for male nurses where a young man, aged 20, could be received without premium?—*Nurse S.*

### Answers.

(158) *Foot Reform* (*Nurse*).—If you mean the makers of the special boots, they are Messrs. Holden, 223, Regent Street, London. Is this the firm you mean? We have returned the postal order you enclosed, as there is no charge made for answering questions in this column. Our rule as to letters you can read in the note above.

(159) *Probationer* (*Juvenile*).—You are much too young for a general hospital. Read "How to Become a Nurse," published by the Scientific Press, 428, Strand.

(160) *Registration* (*Nurse D. F. D.*).—If you mean registration through the Royal British Nurses' Association you should apply to the office, which is at 17, Old Cavendish Street, W.

(161) *Vaccination.*—This is a curious instance of the placid way in which some people look upon law-breaking, so long as the law they wish to break deals only with sanitary matters. We cannot, however, be parties to such proceedings, even to the extent of suggesting "what it will cost" to defy an Act of Parliament.

(162) *Male Nurse* (*Nurse S.*)—There is as yet no school for training male nurses in England.

(163) *Probationer* (*Enquirer*).—You should apply to the matron of the hospitals. You can gain full information from the book, "How to Become a Nurse," by Honnor Morten. Scientific Press, 428, Strand, W.C.

(164) *Probationer* (*E. E. P.*)—You had better apply to the matrons of large county infirmaries. See reply to Enquirer.

### Dress and Uniforms.

By a Matron and Superintendent of Nurses.

### ST. GEORGE'S HOSPITAL.

Most of our readers will join us in admiration of the group which it is our privilege this week to describe. St. George's Hospital prides itself, and justly so, on the neat and becoming costumes of its nursing staff. The centre figure is that of a charge nurse, who wears a dress of black Victoria cord, fitting tightly to the figure and just clearing the ground round the bottom. It is turned up with a hem and sewn on to a band at the waist, with gathers or pleats. The material is not only a very serviceable one, but from the fact of its possessing a smooth surface does not harbour dust, and consequently always looks smart and tidy. A plain white linen apron is worn over the dress, to which a bib is attached with straps that cross behind and fasten at the waist. The cap is of the ever-charming "Sister Dora" shape, made in spotted muslin, and edged with narrow Valenciennes lace. The muslin strings, however, instead of fastening in front, tie under the hair behind. Fine linen cuffs relieve the dress at the wrists, and a stand-up collar fits closely round the throat, giving a neat finish to the neck. The staff nurse and probationer wear a similar uniform, the distinguishing mark consisting in the band which it is the privilege of the former to wear round her left arm. The dress is made of galatea of a soft blue shade, which washes admirably, and is well adapted for wear in a sick ward. The bodice is quite plain, fitting tight to the figure, and buttoned in front. The skirt is full and turned up with a deep hem, and is gathered into a band at the waist into which the bodice is fixed. Over this comes an apron of ample size with bib and straps that cross and fasten behind. The cap is identical in shape with that worn by the charge nurse, differing only in the material, which is plain mull muslin. Linen cuffs and collar complete a singularly attractive costume.

NURSES AT ST. GEORGE'S HOSPITAL.

### Where to Go.

South Place Chapel.—Miss Honnor Morten will speak at a quarter-past eleven a.m., on Sunday, June 16th, on "Life in a London Hospital."

Taunton and Somerset Hospital, Taunton.—The nursing staff purpose getting up a bazaar to raise funds for a tennis court for the use of the nurses. Promises of work, plants, &c., will be gladly received by the Lady Superintendent. Work should be sent in not later than September 14th.

### School for the Blind at York.

(Formerly the Palace of King James I.)

Through the ancient palace garden, in the sunshine and the shadow,
I wandered in the stillness of the summer afternoon,
While through the tender greenness I marked the blossoms falling,
Like hopes born of the sunshine in the happy month of June.
From the tree-tops, swaying gently in the warm south wind of summer,
Came song of thrush and linnet from their hidden nests of green,
And the air was full of music and the heart of tender dreaming,
And undefined longings for the bliss of the unseen.

Beneath the carvèd portal, where kings in royal pageant,
With sounds of martial music, had passed in days of yore,
I paused a moment, musing on the transitory grandeur
Of an earthly pride and glory in the days that are no more.
When through the waiting stillness a child's voice rang out clearly,
As if the birds of summer had taught her how to sing,
And following to its leading I found the "Room of Waiting,"
Where the children "sit in darkness" in the palace of the king.

Without was bloom of summer and the beauty of a season,
When men forget their mourning and the world forgets its cares,
Within, through latticed windows, the clear June sun was falling
In vivid gleams of glory on the children's braided hair.
Sitting patient, all unconscious of the sunshine which enfolds them,
Their white, pathetic faces, all too grave for childish glee;
And I marked their small hands lying clasped in those of one another,
As if clinging through the darkness to the love they could not see.

And cleaving through the sunshine the child voice rang on clearly,
A song of life's young summer when the merry days are long,
With her fair face lifted upward as if her soul would follow
To some enchanted region the swift flight of her song.
Oh, children, little children, my heart is filled with sadness,
Ye cannot see the tears that fall in thinking of your loss;
I, who see the blessed sunshine and the faces that I cherish,
Am weeping for your sorrow and the burden of your cross.

For youth is sweet, my children, but ne'er shall glance of lover
Pierce through the gloom that folds thee and wake thy heart to bliss;
The face of him thou lovest is an unattainèd treasure,
A joy whose ceaseless beauty thy heart must ever miss.
When ye come to woman's stature, and wifehood's loving duties
Teach thee each day more keenly all the sorrow of thy loss,
When motherhood shall bless thee with its presence glad and holy,
Will ye still bear on in patience, nor faint beneath the cross?

When ye hear your kinsfolk saying, "The child is like his father,
Forget-me-nots are not more blue, more lovely than his eyes!"
How will ye bear the burden of a grief no man can utter,
Of the mother-love and yearning that can ne'er be satisfied?
Blind to the rosy beauty of the baby mouth that kisses
The aching eyes that long to see, with cravings deep and wild;
Blind to the lovely dimples in the baby face that presses
Against the heart that hungers for the sweet face of your child.

Oh, summer, joyous summer! oh, human love and gladness!
Why are the young eyes holden that they cannot see thy light;
Why has the Father given unto them so hard a burden,
Why placed about their pathway a never-ending night?
What have they done to merit a youth of vain repining,
A life from which all beauty and all sunshine is denied,
Why should their fathers' sinning hurt the innocent young children,
And fill their hearts with longings that can ne'er be satisfied?

Oh, calmly fell the sunshine, and the deep tones of the organ
Throbbed through the silent chamber and filled mine eyes with tears,
And with one accord the children lifted up their voice together
And sang of that far country beyond the flight of years.
"Oh, sweet and blessed country," sang the children clearly, clearly,
"Our feet shall tread thy radiant streets, our eyes thy glories see"—
And my soul on wings of music floated onward, floated upward,
And heard the Saviour calling, "Little children, come to Me!"

And mine eyes, as in a vision, saw Jerusalem the Golden,
For whose diviner knowledge my doubting heart had sighed,
And I heard a voice from Heaven saying, "Weep not for the children,
They shall arise and, seeing God's face, be satisfied."
Oh, doubting soul, be silent; thy God is King for ever,
His little ones shall never miss the safe and narrow way;
Oh, sweet and blessed country! their eyes shall see thy glory,
And find within thy radiant gates a never-ending day!

Peace be within thy portals, oh, time-worn ancient palace!
Where the angel of His Presence abideth night and day;
Where for a little season the children sit in darkness,
"Until the morning breaketh and the shadows flee away."

M. P. T.

### Appointments.

Wakefield Infectious Hospital, Carr Gate.—Miss Annie Eustace has been appointed matron of this institution. She was trained at the Monsall Hospital, Manchester, and after gaining her certificate remained at the hospital as staff nurse for two and a-half years. In January, 1893, she took charge of a temporary small-pox hospital at Sowerby Bridge, in Yorkshire. She resigned this appointment for one at the Infectious Hospital, Garston, Liverpool, whence she now proceeds to Carr Gate. Her testimonials are excellent, and Miss Eustace was selected from amongst over 40 candidates.

### Minor Appointments.

Durham Cottage Hospital.—Miss Scott, who was trained at the Durham County Hospital, has been appointed Head Nurse to the Durham Cottage Hospital.

Poplar and Stepney Asylum, Bromley, Middlesex.—Miss Beatrice Sisley has been appointed Assistant Matron to the above institution. She was trained at and holds the three certificates of St. Bartholomew's Hospital, and Belvidere City Hospital, Glasgow. She held the post of medical and surgical sister at the Victoria Infirmary, Glasgow, and has for three months filled the matron's post at the same institution.

## Figures and Tables

**Fig. 30 f1:**
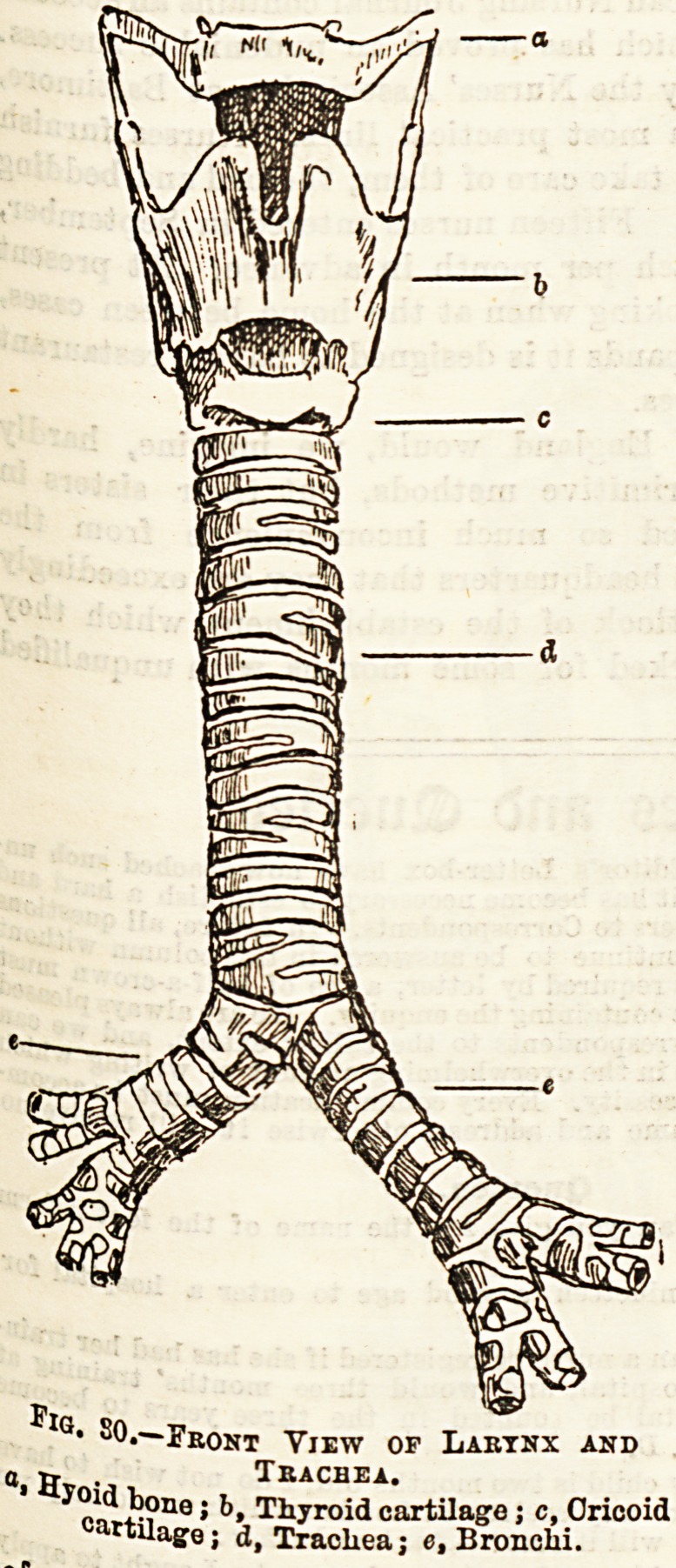


**Fig. 31 f2:**
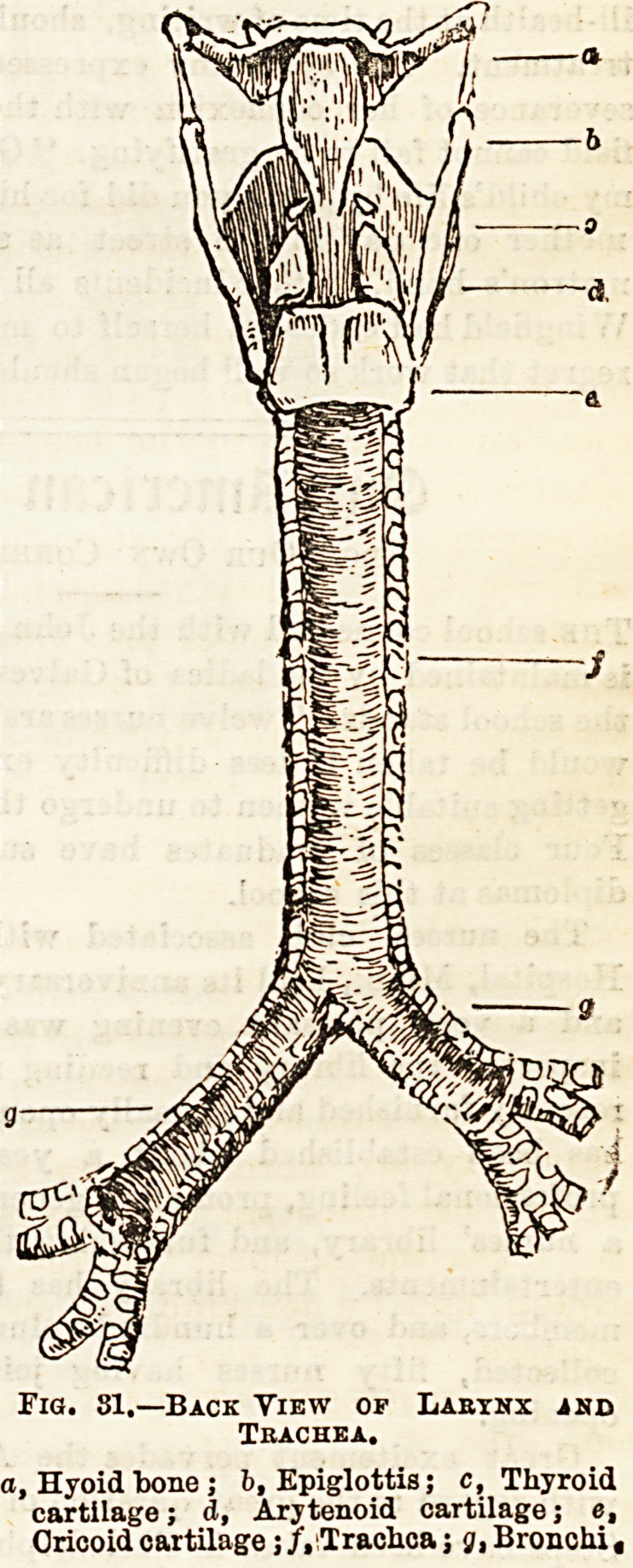


**Figure f3:**